# Molecular causes of congenital anomalies of the kidney and urinary tract (CAKUT)

**DOI:** 10.1186/s40348-021-00112-0

**Published:** 2021-02-24

**Authors:** Stefan Kohl, Sandra Habbig, Lutz T. Weber, Max C. Liebau

**Affiliations:** 1 Department of Pediatrics, Faculty of Medicine, University Hospital Cologne, University of Cologne, Cologne, Germany; 2 Center for Molecular Medicine Cologne (CMMC), University of Cologne, Cologne, Germany

## Abstract

Congenital anomalies of the kidney and urinary tract (CAKUT) occur in 0.5–1/100 newborns and as a group they represent the most frequent cause for chronic kidney failure in children. CAKUT comprise clinically heterogeneous conditions, ranging from mild vesicoureteral reflux to kidney aplasia. Most forms of CAKUT share the pathophysiology of an impaired developmental interaction of the ureteric bud (UB) and the metanephric mesenchyme (MM). In most cases, CAKUT present as an isolated condition. They also may occur as a component in rare multi-organ syndromes. Many CAKUT probably have a multifactorial etiology. However, up to 20% of human patients and > 200 transgenic mouse models have a monogenic form of CAKUT, which has fueled our efforts to unravel molecular kidney (mal-)development. To date, genetic variants in more than 50 genes have been associated with (isolated) CAKUT in humans. In this short review, we will summarize typical imaging findings in patients with CAKUT and highlight recent mechanistic insight in the molecular pathogenesis of monogenic forms of CAKUT.

**Supplementary Information:**

The online version contains supplementary material available at 10.1186/s40348-021-00112-0.

## Congenital anomalies of the kidney and urinary tract (CAKUT) are a major challenge in pediatric nephrology

**Fig. 1:**
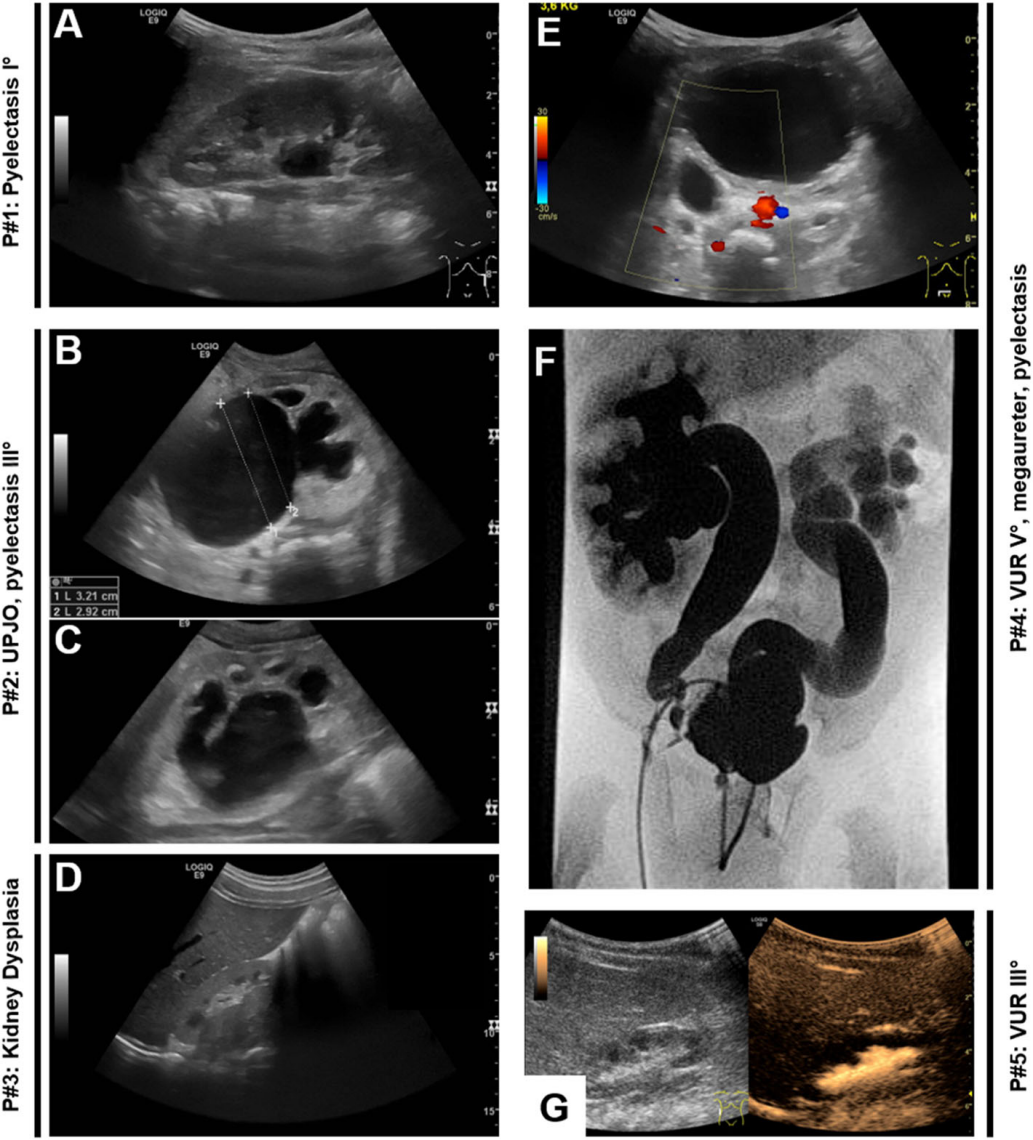
Imaging studies in patients with different CAKUT. (**a**) Mild dilatation of the kidney pelvis (11 mm); (**b** and **c**) severe dilatation of the kidney pelvis (290 mm); and calyxes due to ureteropelvic junction obstruction (UPJO). (**d**) Small and hyperechogenic right kidney without corticomedullary differentiation suggesting kidney dysplasia; (**e**) Dilatation of the right ureter retrovesically; (**f**) voiding cysturetrography (VCUG) demonstrating bilateral VUR V° in the filling phase in the same patient as in **e** (International Reflux Study Committee (1981)). Contrast agent was administered using a transurethral catheter. (**g**) Contrast-enhanced uro-sonography in a patient with VUR III°. Contrast agent is detectable in the right kidney pelvis

The term “CAKUT” (congenital anomalies of the kidney and urinary tract) encompasses a clinically broad spectrum of malformations, including kidney hypoplasia and dysplasia, ureteropelvic junction obstruction (UPJO), primary megaureter, vesicoureteral reflux (VUR), ureterovesical junction obstruction (UVJO), or posterior urethral valves (PUV) (Fig. 1). Together with heart defects, CAKUT are the most common group of malformations in humans, affecting 0.5-1% of newborns according to the EUROCAT database. In most of the cases, CAKUT present as an isolated condition. However, there are numerous rare syndromes that have CAKUT as one component among other malformations (e.g., Fraser syndrome; “cryptophthalmos, syndactyly, and CAKUT;” OMIM #219000). About 40% of children on kidney replacement therapy are suffering from a CAKUT diagnosis, making the CAKUT spectrum the most frequent single cause of chronic kidney failure in children [1].

CAKUT usually are detected by ultrasonography, either prenatally, or due to a complication later in life (Fig. 1), or as an incidental finding. Symptomatic individuals may present with recurrent urinary tract infections, with chronic kidney disease of any stage and at any age, with perinatal kidney failure being the most severe form. Medical care for children with symptomatic CAKUT may be complex and require patient-tailored multidisciplinary approaches. Therapeutic options are restricted to symptomatic treatments that focus on preserving kidney function, preventing urinary tract infections, optimizing quality of life, mitigating symptoms of chronic kidney disease, and, if necessary, providing kidney replacement therapy.

## A minority of patients has monogenic CAKUT

The etiologies of CAKUT often remain elusive, as etiology likely is “multifactorial” in many cases [2]. Recent advances in “disease gene” identification have confirmed that in human patients with CAKUT variants can be found in many genes that are implicated in early kidney development and that are also affected in mouse models with CAKUT [3]. Today, up to 20% of cases may be explained by one of > 50 rare monogenic forms of CAKUT (Supplemental Table 1) [4, 5]. An additional 5-10% of CAKUT cases seem to be caused by larger genetic variations, i.e., copy number variations (CNV) [6, 7]. From a clinical point of view, monogenic CAKUT may be undistinguishable from multifactorial CAKUT. A careful evaluation of family members and thorough screening for involvement of other organs may indicate a monogenic form of CAKUT that could be confirmed by next generation sequencing (NGS)-based genetic testing. Physicians caring for families with CAKUT need to be aware that different individuals from the same family may have different CAKUT phenotypes (“variable expressivity”) and that variant carriers may not be affected (“reduced or incomplete penetrance”), which further complicates the identification of familial cases and counseling of families.

More than 50 “CAKUT-genes” have been described so far (Supplemental Table 1), each of them being responsible for less than 1% of cases in mixed CAKUT cohorts [4, 5]. Variants in almost all of these genes may present as multi-organ syndrome, the most common ones being *HNF1B* (OMIM #13792 Renal cysts and diabetes syndrome) and *PAX2* (OMIM #120330 papillorenal syndrome). However, pathogenic variants in the same genes may also lead to either truly isolated CAKUT, or to CAKUT with only subtle syndromic features that easily can be missed, or to CAKUT with syndromic features presenting later in life (such as cognitive impairment). Consequently, genetic diagnostics in individuals with isolated CAKUT should be rather inclusive. Genetic variants in individuals with CAKUT (and in general) have to be interpreted with caution as recommended by the American College of Medical Genetics (ACMG criteria) [8]. Genetic variants in “CAKUT-genes” should be interpreted by experienced geneticists because multiple variants published as “pathogenic” have turned out to be suspiciously frequent in healthy individuals and thus may merely represent genetic risk factors or even be neutral. For many “CAKUT genes,” functional data supporting causality is lacking (Supplemental Table 1).

## CAKUT arise from a disturbed differentiation/interaction of the ureteric bud and the metanephric mesenchyme

**Fig. 2:**
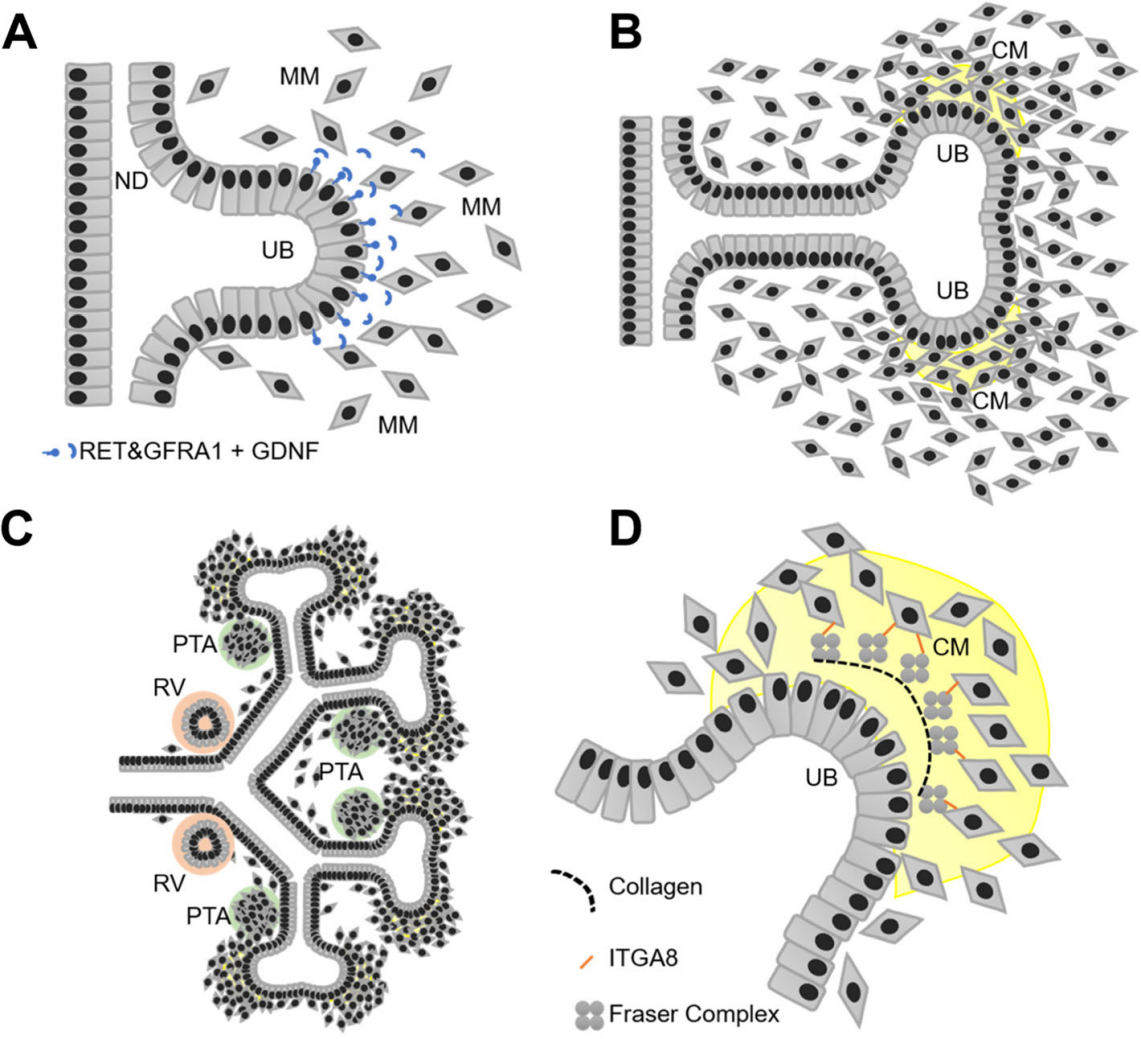
Illustration of ureteric budding, ureteric branching, initiation of nephron formation, and the extra cellular matrix (ECM) interface between UB and MM. **a** Ureteric budding from the nephric duct (“ND”) requires active GDNF/RET/GFRA1 signaling between the ureteric bud (“UB”) and the metanephric mesenchyme (“MM”). **b** Branching of the ureteric bud (“UB”) tip covered by cap mesenchyme (“CM,” yellow) containing nephron progenitor cells (“NPC”). **c** Subpopulations of NPC leave the cap mesenchyme niche, form peritubular aggregates (“PTA,” green), and undergo mesenchymal-epithelial transition to form renal vesicles (“RV,” orange) that will develop further into nephrons (not shown). **d** Illustration of the UB-cap mesenchyme (“CM”)-interface with the “Fraser Complex”-associated extra cellular matrix (ECM). FRAS1 and FREM2 (secreted from the UB) and FREM1 and nephronectin (expressed in the MM/CM) assemble in the extracellular space and bind to the Integrin a8/ß1 receptor located in cells derived from the MM

Despite their clinical divergence, most CAKUT have in common an early mal-development of nephro-uro-genic tissues, i.e., the ureteric bud (UB) and the metanephric mesenchyme (MM). CAKUT of the lower urinary tract (e.g., posterior urethral valves, PUV) likely have a different pathogenesis, which is yet not well understood and will not be the focus of this review. In humans, (meta-)nephrogenesis begins at around gestational week four when the UBs form from the nephric ducts (ND) under the influence of temporospatial molecular cues, e.g., GDNF-RET/GFRA1 signaling (Fig. 2a) [9, 10]. The UBs invade the MM, undergo multiple generations of dichotomous divisions, thereby forming a “UB-tree” that is fully embedded in MM (Fig. 2b and c). Keeping with this analogy, the “UB-trunk” will turn into the ureter, the “UB-branches” will give rise to the upper urinary collecting system, whereas the MM contributes by adjoining the “leaves,” namely, the nephrons (and the kidney/ureter stroma). The centrifugally growing UB-tips are capped by a subpopulation of MM cells, i.e., the cap mesenchyme (Fig. 2b), which harbors a pool of SIX1+/CITED1+ nephron progenitor cells (NPC) [9]. Repeatedly, groups of NPC leave the cap-mesenchyme and become committed to the formation of nephrons by initially forming pretubular aggregates (PTA) (Fig. 2c) [10]. PTA cells undergo a mesenchymal-epithelial transition becoming renal vesicles (RV) (Fig. 2c). RV elongate to form “comma-shaped” and then bend to “S-shaped” bodies, which eventually differentiate into mature, segmented nephrons.

Numerous mouse models with CAKUT phenotypes and identification of monogenic CAKUT in humans have provided mechanistic insight into the molecular pathogenesis of CAKUT. Most of the “CAKUT-genes” can be assigned to one of the following three groups: (I) transcription factors (e.g., *PAX2*, *HNF1B*), (II) signaling molecules (e.g., *ROBO2*), and (III) extracellular matrix (ECM) components of the developing kidney (e.g., *FRAS1*, *FREM2*, *ITGA8*).

## Transcription factors are implicated in CAKUT in humans

The spatiotemporal expression of distinctive sets of transcription factors is essential for proper programming of multi-potent cells, such as SIX1+/CITED1+ positive nephron progenitor cells (NPC), as well as for their descendants, e.g., LHX1+/EMX2+/HNF1B+ cells in pretubular aggregates and renal vesicles (Fig. 2) [9]. Deleterious variants in many of the genes encoding for these kidney developmental transcription factors lead to CAKUT in mice and humans (Supplemental Table 1).

Probably the most recognized example for monogenic CAKUT in human patients among clinicians is *HNF1B* nephro-uro-pathy. Deleterious heterozygous variants in *HNF1B*, such as the frequently reported whole gene deletion (about 50% of cases), lead to heterogeneous malformations of the kidney and/or the urinary tract and/or diabetes mellitus. Commonly, affected patients exhibit ultrasonographic small and bright kidneys with multiple small cysts and different degrees of kidney function impairment. HNF1B is expressed in epithelial cells in the liver, pancreas, and in the kidney, more specifically in UB cells, nephron precursor cells, and in kidney tubules. In developing kidney tubules, HNF1B promotes SOCS3 expression, which plays an important role in tubulogenesis [11]. Tubular dysgenesis in patients with deleterious variants in *HNF1B* is appreciated by the clinical term “*HNF1B* associated Autosomal dominant Tubular Kidney Disease” (HNF1B-ADTKD), which is characterized by chronic kidney disease including hyperuricemia with or without gout, hypokalemia, hypomagnesemia, and polyuria [12]. In a German multicenter childhood registry study for *HNF1B* nephropathy, 87% of probands (54/62) had bilateral kidney dysplasia, whereas the other mentioned symptoms were less frequently observed [13]. The broad expression of HNF1B in UB, liver, and pancreas explains additional extrarenal features, such as “Maturity Onset Diabetes of the Young Type 5” (MODY5) and defects of the urinary collecting system.

Variants in *PAX2* are another well-established example of monogenic CAKUT. Mutations in *PAX2* may cause syndromic CAKUT with ocular anomalies, such as optic nerve coloboma (OMIM # 120330) [14]. PAX2 is expressed in multiple embryonic tissues including the optical disk and the cap mesenchyme (see expression data on www.gudmap.org). In mesenchymal-epithelial transition, PAX2 promotes expression of the podocyte transcription factor WT1. Failure to promote WT1 expression seems to result in kidney dysplasia through impairment of nephron differentiation. Interestingly, patients carrying a deleterious variant in *PAX2* may also present with steroid resistant nephrotic syndrome (SRNS) and focal segmental glomerulosclerosis (FSGS), similar to patients with a deleterious variant in *WT1* [15]. To this notion, patients with PAX2-CAKUT may have early albuminuria, exceeding the level of albuminuria that is expected solely on the basis of chronic kidney disease. Hence, physician should carefully search for eye involvement and consider genetic testing for *PAX2* mutations in patients with CAKUT and early albuminuria.

Other recent examples for transcription factors implicated in CAKUT are *TBX18* and *NRIP1*. TBX18 is essential for differentiation of MM cells into ureter smooth muscle cells. Heterozygous deleterious mutations in *TBX18* prevent smooth muscle differentiation in the ureteral wall. The absence of peristaltic contraction ability causes ureteropelvic junction obstruction (UPJO) and congenital hydronephrosis. This has been discovered and studied in a *Tbx18*+/− mouse model and was later found to also be a rare cause of UPJO and hydronephrosis in human patients [16, 17].

NRIP1 is a co-transcription factor of the retinoic acid receptor RARα. A heterozygous truncating variant in *NRIP1* recently has been identified in a large kindred with different forms of CAKUT (kidney cysts, kidney dysplasia, dilatation of the ureter, and VUR) [18]. A causative role of this variant is supported by *knock-down* of *Nrip1* in *X. laevis* larvae that causes a similar CAKUT phenotype (hydroureter, hydronephrosis, and ureterocele). Interestingly, this discovery is in line with the historic observation that alternations in maternal vitamin A supply during pregnancy increase the risk for CAKUT and thereby provides insight into a possible interplay of genetic and environmental factors [19].

## Signaling molecules are implicated in CAKUT in humans

The presence of specific transcription factors in nephrogenic cells goes hand in hand with expression of specific sets of cell-signaling proteins. Metanephric kidney formation is initiated when mesenchymal cells at the site of ureteric budding secrete the ligand GDNF which finds its receptor RET and co-receptor GFRA1 on epithelial cell of the nephric duct (ND) that will start proliferating to form a bud. Failure in budding leads to kidney agenesis, failure to restrict budding to a single site leads to duplex kidneys. These landmark findings have been studied extensively in mouse models and embryonic kidney organ cultures [20]. Heterozygous variants in *RET* and biallelic variants in *GFRA1* in human patients with (bilateral) kidney agenesis have been described as very rare causes of CAKUT (Supplemental Table 1). Other ligands involved in ureter induction are BMP4 and GREM1, which seem to restrict GDNF signaling to the actual site of budding. Involvement of rare genetic variants in *BMP4* and *GREM1* in CAKUT in humans has been suggested [3, 21].

Another signaling pathway that is involved in nephrogenesis and CAKUT is SLIT2-ROBO2 signaling. The ligand SLIT2 is expressed in MM and cap mesenchyme. The SLIT2 signal is interpreted by epithelial cells of the nephric duct and the UB through the trans-membranous receptor ROBO2 [22]. Variants in *ROBO2*, and likely also variants in *SLIT2* and the downstream small GTPase activator *SRGAP1*, lead to CAKUT (mostly multicystic dysplastic kidneys, MCDK) in humans [23]. SLIT2/ROBO2/SRGAP1 signaling is also present during nephrongenesis in podocytes [24]. Possibly, genetic disturbances in SLIT2/ROBO2 signaling during initial budding lead to ureteral defects, whereas disturbances during nephron formation may lead to kidney hypoplasia or dysplasia.

## Extracellular matrix components are implicated in CAKUT in humans

The cellular compartments of the developing kidney are scaffolded by an extracellular matrix that seems to include a set of proteins which are essential for kidney development, as supported by human and mouse genetic data: The UB epithelial cells express three “Fraser Syndrome” genes *FRAS1*, *FREM2*, and *GRIP1*. Biallelic pathogenic variants in any of these genes lead to ECM defects, e.g., in the skin and developing kidney. Affected human individuals and animal models exhibit a syndromic spectrum of malformations ranging from the most severe Fraser Syndrome (OMIM #219000), to the “mildest” manifestation in form of isolated CAKUT [3]. GRIP1 is essential for secretion of FRAS1 and FREM2 into the extracellular space where they interact with FREM1 and nephronectin (NPNT) in a multi-protein complex (Fig. 2d). This “Fraser Complex” interacts with the Integrin a8/ß1 heterodimer located in the membrane of cells derived from the MM (Fig. 2d). Binding of the “Fraser complex” to Integrin a8/ß1 enables a signaling event that likely acts upstream of GDNF expression in MM, which may explain the kidney agenesis phenotype in mice and humans with deleterious variants in the integrin encoding gene *ITGA8* [25]. In this context, variants in the “CAKUT gene” *HPSE2*, that encodes for a peptidase that cleaves the heparan sulfate side chains to permit the remodeling of the extracellular matrix for cell movement (Supplemental Table 1), are an additional line of evidence for the importance of the nephrogenic ECM in CAKUT.

In summary, CAKUT impose a high burden on affected families and health care. Establishing a molecular genetic diagnosis in patients with monogenic CAKUT helps affected families to understand the etiology of their child’s medical condition. It enables physicians to classify it as accurately as possible and it might open the possibility for a more personalized care. In our pediatric nephrology center, we consider genetic testing in CAKUT in patients with severe CAKUT (i.e., relevant chronic kidney disease), syndromic CAKUT, and/or familial CAKUT. Since CAKUT are a developmental group of conditions with genetic-environmental etiology, there is no causative therapy after birth. Therapeutic strategies focus on prevention and symptomatic treatment, including surgical correction/improvement, avoiding complications, and providing kidney replacement therapy. Studying molecular mechanisms of genes implicated in monogenic CAKUT and animal models has provided insight in nephrogenesis on a cellular and molecular level that may serve as basic research on our journey to growing an artificial, personalized kidney in the future.

## Supplementary Information

**Additional file 1: Supplemental Table 1**. 50 genes that represent monogenic causes/candidate genes of “isolated” CAKUT in humans.

## Data Availability

N/A

## Abbreviations

Array-CGH: Array-based comparative genomic hybridization
CAKUT: Congenital anomalies of the kidney and urinary tract
ECM: Extracellular matrix
MCDK: Multicystic dysplastic kidneys
MM: Metanephric mesenchyme
ND: Nephric duct
UB: Ureteric bud
UPJO: Ureteropelvic junction obstruction
UVJO: Ureterovesical junction obstruction
VUR: Vesicoureteral reflux
WES: Whole exome sequencing
