# Supplementary material for: Molecular causes of congenital anomalies of the kidney and urinary tract (CAKUT)
Source: Mol Cell Pediatr. 2021 Feb 24;8:2. doi: 10.1186/s40348-021-00112-0 (PMC7904997; doi:10.1186/s40348-021-00112-0)
Supplement: Supplementary file 1 — Additional file 1: Supplemental Table 1. 50 genes that represent monogenic causes/candidate genes of “isolated” CAKUT in humans. [file 40348_2021_112_MOESM1_ESM.docx]

Molecular causes of Congenital Anomalies of the Kidney and Urinary Tract (CAKUT)

Stefan Kohl, Sandra Habbig, Lutz T. Weber, Max C. Liebau

Supplemental Table 1: 50 genes that represent monogenic causes/candidate genes of “isolated” CAKUT in humans

| **Gene** | **Protein** | **Level of evidence^1^** | **OMIM^2^** | **Ref.** |
| --- | --- | --- | --- | --- |
| **Autosomal recessive (n = 16)** | | | | |
| *ACE* | Angiotensin I–converting enzyme | G(+), R(+), A (+) | [267430](https://www.omim.org/entry/267430) | [1] |
| *AGT* | Angiotensinogen | G(+), R(+), A (+) | [267430](https://www.omim.org/entry/267430) | [1] |
| *AGTR1* | Angiotensin II receptor, type 1 | G(+), R(0), A (+) | [267430](https://www.omim.org/entry/267430) | [1] |
| *CHRM3* | Muscarinic acetylcholine receptor M3 | G(+), R(+), A (+) | [100100](https://www.omim.org/entry/100100) | [2] |
| *FGF20* | Fibroblast growth factor 20 | G(+), R(0), A (+) | [615721](https://www.omim.org/entry/615721) | [3] |
| *FRAS1* | ECM protein FRAS1 | G(+), R(+), A (+) | [219000](https://www.omim.org/entry/219000) | [4, 5] |
| *FREM1* | FRAS1-related ECM protein 1 | G(0), R(0), A (+) | [248450](https://www.omim.org/entry/248450) [608980](https://www.omim.org/entry/608980) | [4] |
| *FREM2* | FRAS1-related ECM protein 2 | G(0), R(0), A (+) | [617666](https://www.omim.org/entry/617666) [123570](https://www.omim.org/entry/123570) | [4] |
| *GFRA1* | GDNF family receptor alpha-1 | G(+), R(0), A (+) | n/a | [6] |
| *GRIP1* | Glutamate receptor interacting protein 1 | G(0), R(0), A (+) | [617667](https://www.omim.org/entry/617667) | [4] |
| *HOXA11* | Homeobox protein Hox-A11 | G(0), R(0), A (0) | n/a | [7] |
| *HPSE2* | Heparanase 2 | G(+), R(+), A (+) | [236730](https://www.omim.org/entry/236730) | [8] |
| *ITGA8* | Integrin alpha-8 | G(+), R(+), A (+) | [191830](https://www.omim.org/entry/191830) | [9] |
| *REN* | Renin | G(+), R(+), A (+) | [267430](https://www.omim.org/entry/267430) | [1] |
| *TRAP1* | Heat shock protein 75 kDa, mitochondrial | G(0), R(0), A (0) | n/a | [10] |
| *VWA2* | von Willebrand factor A domain-containing protein 2 | G(0), R(0), A (1) | n/a | [11] |
|  |  |  |  |  |
| **Autosomal dominant (n = 33)** | | | | |
| *BMP4* | Bone morphogenetic protein 4 | G(0), R(0), A (+) | n/a | [12] |
| *BNC2* | Zinc finger protein basonuclin-2 | G(0), R(0), A (+) | [618612](https://www.omim.org/entry/618612) | [13] |
| *CHD1L* | Chromodomain-helicase-DNA-binding protein 1-like | G(0), R(1), A (0) | n/a | [14, 15] |
| *COL4A1* | Collagen alpha-1(IV) chain | G(0), R(0), A (0) | n/a | [16] |
| *CRKL* | Crk-like protein | G(0), R(0), A (+) | n/a | [17] |
| *DSTYK* | Dual serine/threonine and tyrosine protein kinase | G(0), R(0), A (+) | [610805](https://www.omim.org/entry/610805) | [18] |
| *EYA1* | Eyes absent homolog 1 | G(+), R(+), A (+) | [113650](https://www.omim.org/entry/113650) | [19] |
| *FOXC1* | Forkhead box protein C1 | G(0), R(0), A (+) | n/a | [20] |
| *GATA3* | Trans-acting T-cell-specific transcription factor GATA-3 | G(+), R(+), A (+) | [146255](https://www.omim.org/entry/146255) | [21] |
| *GREB1L* | GREB1-like protein | G(+), R(+), A (+) | [617805](https://www.omim.org/entry/617805) | [22, 23] |
| *HNF1B* | Hepatocyte nuclear factor 1-beta | G(+), R(+), A (+) | [137920](https://www.omim.org/entry/137920) | [24, 25] |
| *MUC1* | Mucin 1 | G(+), R(+), A (?) | [174000](https://www.omim.org/entry/174000) | [26] |
| *NRIP1* | Nuclear receptor-interacting protein 1 | G(+), R(0), A (+) | [618270](https://www.omim.org/entry/618270) | [27] |
| *PAX2* | Paired box protein Pax-2 | G(+), R(+), A (+) | [120330](https://www.omim.org/entry/120330) [616002](https://www.omim.org/entry/616002) | [28, 29] |
| *PBX1* | Pre-B-cell leukemia transcription factor 1 | G(+), R(+), A (+) | [617641](https://www.omim.org/entry/617641) | [30–32] |
| *REN* | Renin | G(+), R(+), A (+) | [613092](https://www.omim.org/entry/613092) | [33] |
| *RET* | Proto-oncogene tyrosine-protein kinase receptor Ret | G(0), R(+), A (+) | n/a | [34] |
| *ROBO2* | Roundabout homolog 2 | G(+), R(+), A (+) | [610878](https://www.omim.org/entry/610878) | [35] |
| *SALL1* | Sal-like protein 1 | G(+), R(+), A (+) | [107480](https://www.omim.org/entry/107480) | [36] |
| *SIX2* | Homeobox protein SIX2 | G(0), R(+), A (+) | n/a | [12] |
| *SIX5* | Homeobox protein SIX5 | G(0), R(0), A (0) | [610896](https://www.omim.org/entry/610896) | [37, 38] |
| *SLIT2* | Slit homolog 2 protein | G(0), R(0), A (0) | n/a | [39] |
| *SON* | Protein SON | G(1), R(1), A (1) | [617140](https://www.omim.org/entry/617140) | [40] |
| *SOX17* | Transcription factor SOX-17 | G(0), R(0), A (0) | [613674](https://www.omim.org/entry/613674) | [41] |
| *SRGAP1* | SLIT-ROBO Rho GTPase-activating protein 1 | G(0), R(0), A (0) | n/a | [39] |
| *TBC1D1* | TBC1 domain family member 1 | G(0), R(0), A (+) | n/a | [42] |
| *TBX18* | T-box transcription factor TBX18 | G(+), R(0), A (+) | [143400](https://www.omim.org/entry/143400) | [43] |
| *TBX6* | T-box transcription factor TBX6 | G(+), R(0), A (+) | n/a | [44, 45] |
| *TNXB* | Tenascin-X | G(+), R(0), A (0) | [615963](https://www.omim.org/entry/615963) | [46] |
| *UMOD* | Uromodulin | G(+), R(+), A (+) | [609886](https://www.omim.org/entry/609886)  [162000](https://www.omim.org/entry/162000)  [603860](https://www.omim.org/entry/603860) | [47] |
| *UPK3A* | Uroplakin-3a | G(+), R(0), A (+) | n/a | [48] |
| *WNT4* | Protein Wnt-4 | G(0), R(0), A (+) | n/a | [49] |
| *ZMYM2* | Zinc finger MYM-type protein 2 | G(+), R(0), A (+) | n/a | [50] |
|  |  |  |  |  |
| **X-linked recessive (n = 1)** | | | | |
| *ANOS1* | Anosmin-1 | G(+), R(+), A (+) | [308700](https://www.omim.org/entry/308700) | [51] |

**^1^**Level of evidence as determined by three parameters:
(**G**) “Genetics”: Convincing clinical genetic data (i.e. convincing segregation in large kindreds, > 3 unrelated affected individuals, *de-novo* variants) are available in the original publication, on OMIM.org, or the HGMD database (http://www.hgmd.cf.ac.uk/),
(**R**) “Replication”: Additional unrelated individuals published by at least one other laboratory, and
(**A**) “Animal model”: Animal model data supporting causality of CAKUT phenotype.
*Comment: The assessment of the level of evidence was conducted applying objective criteria. A low level of evidence in this assessment, of course, has limitations, one being the extreme rarity of these conditions.*

**^2^OMIM** accession numbers referring to a CAKUT phenotype.

References:

1. Gribouval O, Gonzales M, Neuhaus T, et al (2005) Mutations in genes in the renin-angiotensin system are associated with autosomal recessive renal tubular dysgenesis. Nat Genet 37:964–968. https://doi.org/10.1038/ng1623

2. Weber S, Thiele H, Mir S, et al (2011) Muscarinic Acetylcholine Receptor M3 Mutation Causes Urinary Bladder Disease and a Prune-Belly-like Syndrome. Am J Hum Genet 89:668–674. https://doi.org/10.1016/j.ajhg.2011.10.007

3. Barak H, Huh S-H, Chen S, et al (2012) FGF9 and FGF20 maintain the stemness of nephron progenitors in mice and man. Dev Cell 22:1191–1207. https://doi.org/10.1016/j.devcel.2012.04.018

4. Kohl S, Hwang D-Y, Dworschak GC, et al (2014) Mild recessive mutations in six Fraser syndrome-related genes cause isolated congenital anomalies of the kidney and urinary tract. J Am Soc Nephrol 25:1917–1922. https://doi.org/10.1681/ASN.2013101103

5. Ahn YH, Lee C, Kim NKD, et al (2020) Targeted Exome Sequencing Provided Comprehensive Genetic Diagnosis of Congenital Anomalies of the Kidney and Urinary Tract. J Clin Med 9:. https://doi.org/10.3390/jcm9030751

6. Arora V, Khan S, El-Hattab AW, et al (2020) Biallelic Pathogenic GFRA1 Variants Cause Autosomal Recessive Bilateral Renal Agenesis. J Am Soc Nephrol. https://doi.org/10.1681/ASN.2020040478

7. Saygili S, Atayar E, Canpolat N, et al (2020) A homozygous HOXA11 variation as a potential novel cause of autosomal recessive CAKUT. Clin Genet. https://doi.org/10.1111/cge.13813

8. Pang J, Zhang S, Yang P, et al (2010) Loss-of-function mutations in HPSE2 cause the autosomal recessive urofacial syndrome. Am J Hum Genet 86:957–962. https://doi.org/10.1016/j.ajhg.2010.04.016

9. Humbert C, Silbermann F, Morar B, et al (2014) Integrin alpha 8 recessive mutations are responsible for bilateral renal agenesis in humans. Am J Hum Genet 94:288–294. https://doi.org/10.1016/j.ajhg.2013.12.017

10. Saisawat P, Kohl S, Hilger AC, et al (2014) Whole-exome resequencing reveals recessive mutations in TRAP1 in individuals with CAKUT and VACTERL association. Kidney Int 85:1310–1317. https://doi.org/10.1038/ki.2013.417

11. van der Ven AT, Kobbe B, Kohl S, et al (2018) A homozygous missense variant in VWA2, encoding an interactor of the Fraser-complex, in a patient with vesicoureteral reflux. PLoS ONE 13:e0191224. https://doi.org/10.1371/journal.pone.0191224

12. Weber S, Taylor JC, Winyard P, et al (2008) SIX2 and BMP4 mutations associate with anomalous kidney development. J Am Soc Nephrol 19:891–903. https://doi.org/10.1681/ASN.2006111282

13. Kolvenbach CM, Dworschak GC, Frese S, et al (2019) Rare Variants in BNC2 Are Implicated in Autosomal-Dominant Congenital Lower Urinary-Tract Obstruction. Am J Hum Genet 104:994–1006. https://doi.org/10.1016/j.ajhg.2019.03.023

14. Brockschmidt A, Chung B, Weber S, et al (2012) CHD1L: a new candidate gene for congenital anomalies of the kidneys and urinary tract (CAKUT). Nephrol Dial Transplant 27:2355–2364. https://doi.org/10.1093/ndt/gfr649

15. Hwang D-Y, Dworschak GC, Kohl S, et al (2014) Mutations in 12 known dominant disease-causing genes clarify many congenital anomalies of the kidney and urinary tract. Kidney Int 85:1429–1433. https://doi.org/10.1038/ki.2013.508

16. Kitzler TM, Schneider R, Kohl S, et al (2019) COL4A1 mutations as a potential novel cause of autosomal dominant CAKUT in humans. Hum Genet 138:1105–1115. https://doi.org/10.1007/s00439-019-02042-4

17. Lopez-Rivera E, Liu YP, Verbitsky M, et al (2017) Genetic Drivers of Kidney Defects in the DiGeorge Syndrome. N Engl J Med 376:742–754. https://doi.org/10.1056/NEJMoa1609009

18. Sanna-Cherchi S, Sampogna RV, Papeta N, et al (2013) Mutations in DSTYK and dominant urinary tract malformations. N Engl J Med 369:621–629. https://doi.org/10.1056/NEJMoa1214479

19. Abdelhak S, Kalatzis V, Heilig R, et al (1997) A human homologue of the Drosophila eyes absent gene underlies branchio-oto-renal (BOR) syndrome and identifies a novel gene family. Nat Genet 15:157–164. https://doi.org/10.1038/ng0297-157

20. Wu C-HW, Mann N, Nakayama M, et al (2020) Phenotype expansion of heterozygous FOXC1 pathogenic variants toward involvement of congenital anomalies of the kidneys and urinary tract (CAKUT). Genet Med 22:1673–1681. https://doi.org/10.1038/s41436-020-0844-z

21. Van Esch H, Groenen P, Nesbit MA, et al (2000) GATA3 haplo-insufficiency causes human HDR syndrome. Nature 406:419–422. https://doi.org/10.1038/35019088

22. Brophy PD, Rasmussen M, Parida M, et al (2017) A Gene Implicated in Activation of Retinoic Acid Receptor Targets Is a Novel Renal Agenesis Gene in Humans. Genetics 207:215–228. https://doi.org/10.1534/genetics.117.1125

23. Sanna-Cherchi S, Khan K, Westland R, et al (2017) Exome-wide Association Study Identifies GREB1L Mutations in Congenital Kidney Malformations. Am J Hum Genet 101:789–802. https://doi.org/10.1016/j.ajhg.2017.09.018

24. Horikawa Y, Iwasaki N, Hara M, et al (1997) Mutation in hepatocyte nuclear factor-1 beta gene (TCF2) associated with MODY. Nat Genet 17:384–385. https://doi.org/10.1038/ng1297-384

25. Ulinski T, Lescure S, Beaufils S, et al (2006) Renal phenotypes related to hepatocyte nuclear factor-1beta (TCF2) mutations in a pediatric cohort. J Am Soc Nephrol 17:497–503. https://doi.org/10.1681/ASN.2005101040

26. Kirby A, Gnirke A, Jaffe DB, et al (2013) Mutations causing medullary cystic kidney disease type 1 lie in a large VNTR in MUC1 missed by massively parallel sequencing. Nat Genet 45:299–303. https://doi.org/10.1038/ng.2543

27. Vivante A, Mann N, Yonath H, et al (2017) A Dominant Mutation in Nuclear Receptor Interacting Protein 1 Causes Urinary Tract Malformations via Dysregulation of Retinoic Acid Signaling. J Am Soc Nephrol 28:2364–2376. https://doi.org/10.1681/ASN.2016060694

28. Sanyanusin P, Schimmenti LA, McNoe LA, et al (1995) Mutation of the PAX2 gene in a family with optic nerve colobomas, renal anomalies and vesicoureteral reflux. Nat Genet 9:358–364. https://doi.org/10.1038/ng0495-358

29. Vivante A, Chacham OS, Shril S, et al (2019) Dominant PAX2 mutations may cause steroid-resistant nephrotic syndrome and FSGS in children. Pediatr Nephrol 34:1607–1613. https://doi.org/10.1007/s00467-019-04256-0

30. Heidet L, Morinière V, Henry C, et al (2017) Targeted Exome Sequencing Identifies PBX1 as Involved in Monogenic Congenital Anomalies of the Kidney and Urinary Tract. J Am Soc Nephrol 28:2901–2914. https://doi.org/10.1681/ASN.2017010043

31. Riedhammer KM, Siegel C, Alhaddad B, et al (2017) Identification of a Novel Heterozygous De Novo 7-bp Frameshift Deletion in PBX1 by Whole-Exome Sequencing Causing a Multi-Organ Syndrome Including Bilateral Dysplastic Kidneys and Hypoplastic Clavicles. Front Pediatr 5:251. https://doi.org/10.3389/fped.2017.00251

32. Slavotinek A, Risolino M, Losa M, et al (2017) De novo, deleterious sequence variants that alter the transcriptional activity of the homeoprotein PBX1 are associated with intellectual disability and pleiotropic developmental defects. Hum Mol Genet 26:4849–4860. https://doi.org/10.1093/hmg/ddx363

33. Stibůrková B, Majewski J, Hodanová K, et al (2003) Familial juvenile hyperuricaemic nephropathy (FJHN): linkage analysis in 15 families, physical and transcriptional characterisation of the FJHN critical region on chromosome 16p11.2 and the analysis of seven candidate genes. Eur J Hum Genet 11:145–154. https://doi.org/10.1038/sj.ejhg.5200937

34. Skinner MA, Safford SD, Reeves JG, et al (2008) Renal aplasia in humans is associated with RET mutations. Am J Hum Genet 82:344–351. https://doi.org/10.1016/j.ajhg.2007.10.008

35. Lu W, van Eerde AM, Fan X, et al (2007) Disruption of ROBO2 is associated with urinary tract anomalies and confers risk of vesicoureteral reflux. Am J Hum Genet 80:616–632. https://doi.org/10.1086/512735

36. Kohlhase J, Wischermann A, Reichenbach H, et al (1998) Mutations in the SALL1 putative transcription factor gene cause Townes-Brocks syndrome. Nat Genet 18:81–83. https://doi.org/10.1038/ng0198-81

37. Hoskins BE, Cramer CH, Silvius D, et al (2007) Transcription factor SIX5 is mutated in patients with branchio-oto-renal syndrome. Am J Hum Genet 80:800–804. https://doi.org/10.1086/513322

38. Krug P, Morinière V, Marlin S, et al (2011) Mutation screening of the EYA1, SIX1, and SIX5 genes in a large cohort of patients harboring branchio-oto-renal syndrome calls into question the pathogenic role of SIX5 mutations. Hum Mutat 32:183–190. https://doi.org/10.1002/humu.21402

39. Hwang D-Y, Kohl S, Fan X, et al (2015) Mutations of the SLIT2-ROBO2 pathway genes SLIT2 and SRGAP1 confer risk for congenital anomalies of the kidney and urinary tract. Hum Genet 134:905–916. https://doi.org/10.1007/s00439-015-1570-5

40. Kim J-H, Park EY, Chitayat D, et al (2019) SON haploinsufficiency causes impaired pre-mRNA splicing of CAKUT genes and heterogeneous renal phenotypes. Kidney Int 95:1494–1504. https://doi.org/10.1016/j.kint.2019.01.025

41. Gimelli S, Caridi G, Beri S, et al (2010) Mutations in SOX17 are associated with congenital anomalies of the kidney and the urinary tract. Hum Mutat 31:1352–1359. https://doi.org/10.1002/humu.21378

42. Kosfeld A, Kreuzer M, Daniel C, et al (2016) Whole-exome sequencing identifies mutations of TBC1D1 encoding a Rab-GTPase-activating protein in patients with congenital anomalies of the kidneys and urinary tract (CAKUT). Hum Genet 135:69–87. https://doi.org/10.1007/s00439-015-1610-1

43. Vivante A, Kleppa M-J, Schulz J, et al (2015) Mutations in TBX18 Cause Dominant Urinary Tract Malformations via Transcriptional Dysregulation of Ureter Development. Am J Hum Genet 97:291–301. https://doi.org/10.1016/j.ajhg.2015.07.001

44. Yang N, Wu N, Dong S, et al (2020) Human and mouse studies establish TBX6 in Mendelian CAKUT and as a potential driver of kidney defects associated with the 16p11.2 microdeletion syndrome. Kidney Int. https://doi.org/10.1016/j.kint.2020.04.045

45. Dong S, Wang C, Li X, et al (2019) Noncoding rare variants of TBX6 in congenital anomalies of the kidney and urinary tract. Mol Genet Genomics 294:493–500. https://doi.org/10.1007/s00438-018-1522-6

46. Gbadegesin RA, Brophy PD, Adeyemo A, et al (2013) TNXB mutations can cause vesicoureteral reflux. J Am Soc Nephrol 24:1313–1322. https://doi.org/10.1681/ASN.2012121148

47. Hart TC, Gorry MC, Hart PS, et al (2002) Mutations of the UMOD gene are responsible for medullary cystic kidney disease 2 and familial juvenile hyperuricaemic nephropathy. J Med Genet 39:882–892. https://doi.org/10.1136/jmg.39.12.882

48. Jenkins D, Bitner-Glindzicz M, Malcolm S, et al (2005) De novo Uroplakin IIIa heterozygous mutations cause human renal adysplasia leading to severe kidney failure. J Am Soc Nephrol 16:2141–2149. https://doi.org/10.1681/ASN.2004090776

49. Vivante A, Mark-Danieli M, Davidovits M, et al (2013) Renal hypodysplasia associates with a WNT4 variant that causes aberrant canonical WNT signaling. J Am Soc Nephrol 24:550–558. https://doi.org/10.1681/ASN.2012010097

50. Connaughton DM, Dai R, Owen DJ, et al (2020) Mutations of the Transcriptional Corepressor ZMYM2 Cause Syndromic Urinary Tract Malformations. Am J Hum Genet 107:727–742. https://doi.org/10.1016/j.ajhg.2020.08.013

51. Bick D, Franco B, Sherins RJ, et al (1992) Brief report: intragenic deletion of the KALIG-1 gene in Kallmann’s syndrome. N Engl J Med 326:1752–1755. https://doi.org/10.1056/NEJM199206253262606
